# Correlation between the radial artery resistance index and the systemic vascular resistance index: a cross-sectional study

**DOI:** 10.1186/s13089-024-00379-0

**Published:** 2024-05-27

**Authors:** Edith Elianna Rodríguez Aparicio, David Fernando Almanza Hernández, Cristhian Rubio Ramos, María Paula Moreno Knudsen, David Rene Rodriguez Lima

**Affiliations:** 1 Critical and Intensive Care Medicine, Escuela de Medicina y Ciencias de la Salud, Universidad del Rosario, Bogotá, Colombia; 2 Critical and Intensive Care Medicine, Hospital Universitario Mayor-Méderi, Bogotá, Colombia; 3 Anesthesiology Department, Hospital Universitario Mayor-Méderi, Bogotá, Colombia; 4 Grupo de Investigación Clínica, Escuela de Medicina y Ciencias de la Salud, Universidad del Rosario, Bogotá, Colombia

**Keywords:** Hemodynamic assessment, Systemic vascular resistance, Resistance index, Ultrasound

## Abstract

**Introduction:**

Ultrasound measurement of the radial resistance index (RRI) in the anatomical snuffbox has been proposed as a useful method for assessing the systemic vascular resistance index (SVRI). This study aims to establish the correlation between SVRI measured by pulmonary artery catheter (PAC) and RRI.

**Methods:**

A cross-sectional study included all consecutive patients undergoing postoperative (POP) cardiac surgery with hemodynamic monitoring using PAC. Hemodynamic assessment was performed using PAC, and RRI was measured with ultrasound in the anatomical snuffbox. The Pearson correlation test was used to establish the correlation between RRI and SVRI measured using PAC. Hemodynamic behavior concerning RRI with a cutoff point of 1.1 (described to estimate under SVRI) was examined. Additionally, consistency between two evaluators was assessed for RRI using the intraclass correlation coefficient and Bland-Altman analysis.

**Results:**

A total of 35 measurements were obtained. The average cardiac index (CI) was 2.73 ± 0.64 L/min/m², and the average SVRI was 1967.47 ± 478.33 dyn·s·m²/cm^5^. The correlation between RRI and SVRI measured using PAC was 0.37 [95% CI 0.045–0.62]. The average RRI was 0.94 ± 0.11. RRI measurements > 1.1 had a mean SVRI of 2120.79 ± 673.48 dyn·s·m²/cm^5^, while RRI measurements ≤ 1.1 had a mean SVRI of 1953.1 ± 468.17 dyn·s·m²/cm^5^ (*p* = 0.62). The consistency between evaluators showed an intraclass correlation coefficient of 0.88 [95% CI 0.78–0.93], and Bland-Altman analysis illustrated adequate agreement of RRI evaluators.

**Conclusions:**

For patients in cardiac surgery POP, the correlation between the SVRI measured using PAC and the RRI measured in the anatomical snuffbox is low. Using the RRI as a SVRI estimator for patients is not recommended in this clinical scenario.

## Introduction

Evaluating hemodynamics in critically ill patients presents a significant challenge. The use of pulmonary artery catheter (PAC) was described in 1967 by the physicians Jeremy Swan and William Ganz [1]. Since then, hemodynamic values obtained using the pulmonary artery catheter (PAC) have been regarded as the gold standard for defining “shock” [2]. The hemodynamic variables defining shock include the cardiac index (CI), filling pressures, and systemic vascular resistance (SVR). The estimation of SVR is conducted following the principle established by Stefadouros et al. [3], who, in 1973, developed the mathematical proposal currently employed with the PAC.

Bedside ultrasound emerges as a simple, accessible, and cost-effective tool for hemodynamic monitoring, showing good correlation and approximation to the PAC [4, 5]. Ultrasound allows estimation of CI and filling pressures [6–10], although there is limited evidence for calculating SVR using this method. Drawing on the Windkessel principle and the behavior of the arterial compartment, the assessment of resistance indexes (RI) in different vascular beds has been proposed as a potential estimator of systemic vascular resistance index (SVRI) [11–13]. In cardiac surgery, a previous study demonstrated an acceptable correlation between the RI measured in the anatomical snuffbox in the radial artery and SVRI [13], but this correlation has not been consistently replicated in other studies.

This study aims to establish the existing correlation between SVRI measured using the PAC and the radial resistance index (RRI) at the anatomical snuffbox level in patients undergoing postoperative cardiac surgery (POP).

## Methods

### Design

An observational cross-sectional study was conducted, and a sample size of 19 patients was calculated to achieve 80% power in detecting the correlation magnitude between the RRI and the SVRI with a 95% confidence interval.

### Patients / population

Patients in POP cardiac surgery admitted to the intensive care unit, who had undergone hemodynamic monitoring using a PAC from the operating room, were included in the study. Exclusion criteria comprised patients with symptomatic chronic occlusive arterial disease, bilateral arteriovenous fistulas, technical impossibility for measurement, or bilateral absence of upper extremities. The collection and measurement protocol received approval from the Ethics Committee of Universidad del Rosario (DVO005 2149-CV1642) and were carried out at a tertiary-level hospital in Bogotá, Colombia.

### Intervention / measurement

**Fig. 1:**
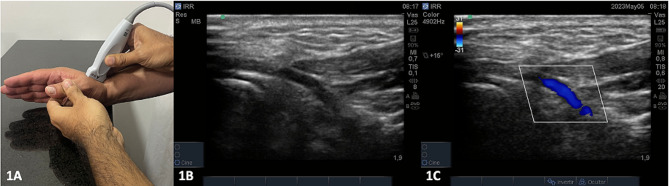
Technique for the measurement of the radial resistance index. **A**. Transducer placing. **B**. B Mode examination of the dorsal branch of the radial artery in the anatomical snuffbox. **C**. Color Doppler mode examination of the dorsal branch of the radial artery in the anatomical snuffbox

**Fig. 2:**
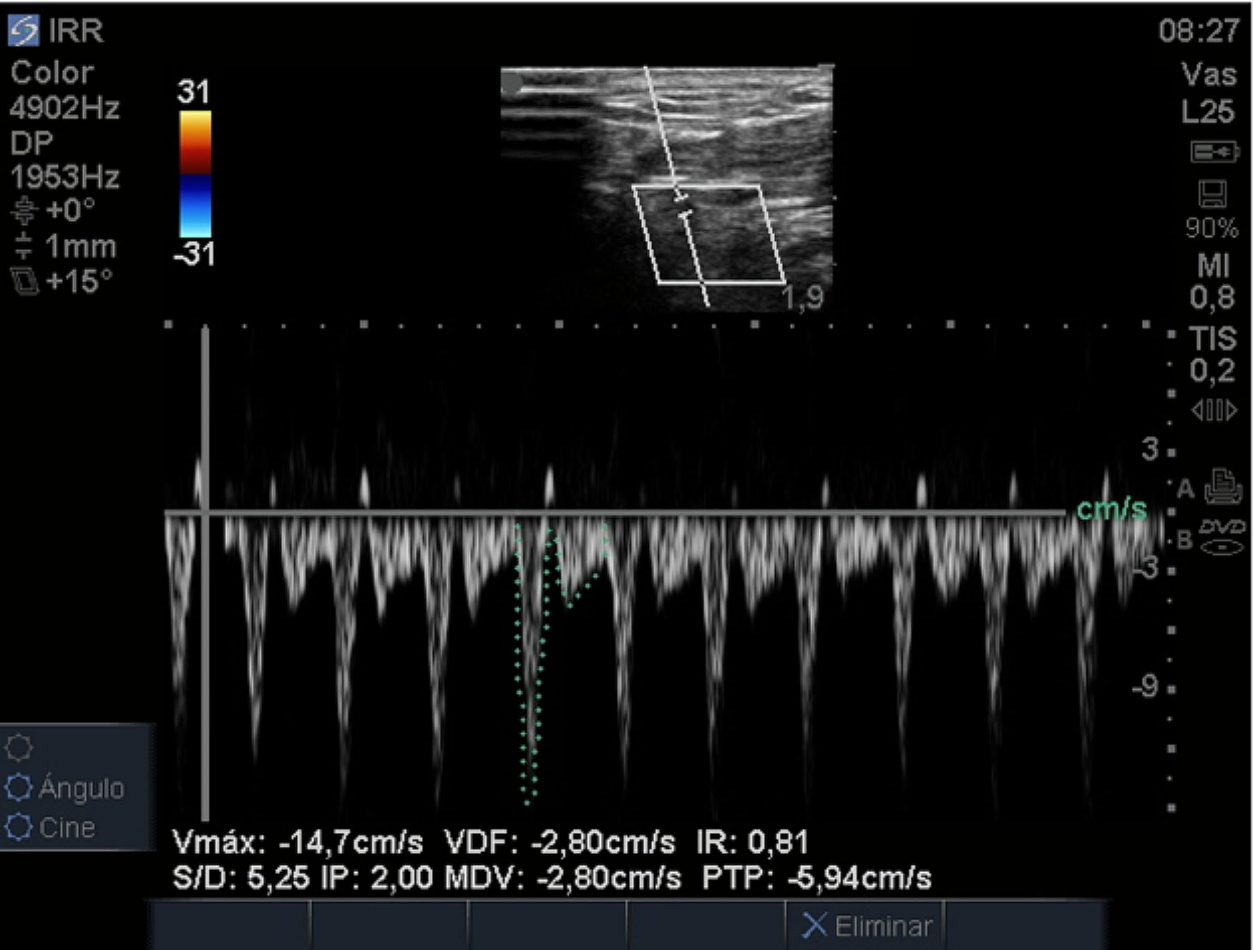
Spectral wave of the dorsal branch of the radial artery in the anatomical snuffbox and calculation of the radial resistance index

Ultrasound measurements were performed by two senior intensive care residents under the supervision of an intensive care physician with over 5 years of experience in cardiovascular intensive care and critical ultrasound. The supervising physician provided training and reviewed the quality of all study images. Ultrasound measurements were taken immediately after hemodynamic measurements with the PAC. For RRI assessment, the transducer was positioned at the level of the anatomical snuffbox with the indicator pointing towards the wrist of the patient (Fig. 1a). The dorsal branch of the radial artery was visualized in mode B and Doppler color (Fig. 1b and c). Using the pulsed Doppler mode, the spectral image of the arterial flow in this area was assessed to obtain the maximum systolic velocity (Vmax) and the minimum diastolic velocity (Vmin). The RRI was calculated using the Pourcelott formula (Vmax-Vmin/Vmax) (Fig. 2) [14]. All images were acquired using a Sonosite Turbo® ultrasound scanner.

The study described the following demographic and clinical characteristics: age, sex, body mass index (BMI), type of cardiac surgery, history of peripheral arterial disease, core temperature, and the use of vasoactive drugs (vasopressors, inotropes, and vasodilators) at the time of measurement. Peripheral arterial disease was defined as the presence of Doppler findings indicating arterial stenosis > 50% in lower or upper limbs from previous surgery. The vasopressors used were norepinephrine and vasopressin, inotropic drugs included milrinone, dobutamine, and levosimendan, and the vasodilator used was nitroglycerin. The hemodynamics measured using the PAC and RRI were also detailed.

### Statical analysis

For continuous variables, mean and standard deviation (SD) or median and interquartile range (IQR) were reported based on the nature of data distribution. Categorical variables were described using absolute and relative frequencies. A bivariate analysis related to RRI ≤ 1.1 and > 1.1 was presented, as this cutoff point has been described in the literature to discriminate patients with altered SVRI [12]. To compare both groups, either the t-test or the Mann-Whitney test was used, depending on the marginal normality.

To assess the association between SVRI and RRI, a simple linear regression model was applied, and the results were visualized through a scatter plot. The r^2^ was calculated using the Pearson method to establish the correlation between RRI and SVRI measured with PAC. In an exploratory manner, it is suggested that the use of vasopressors and/or inotropes, along with temperature, may alter the association between RRI and SVRI due to their effect on peripheral vasculature. Multiple linear regression models were employed to assess this, providing coefficients and statistical significance. Graphical representations of these models were also provided.

The consistency between evaluators was evaluated using the intraclass correlation coefficient and Bland-Altman analysis.

## Results

A total of 35 measurements were obtained from 22 patients. Table 1 presents the general characteristics of the patients and the hemodynamics measured using the PAC and RRI. The average cardiac index (CI) was 2.73 ± 0.64 L/min/m² of total body surface area (TBSA), the average pulmonary artery occlusion pressure (PAOP) was 17 ± 4.82 millimeters of mercury (mmHg), the mean central venous pressure (CVP) was 12.94 ± 3.2 mmHg, and the average systemic vascular resistance index (SVRI) was 1967.47 ± 478.33 dyn·s·m²/cm^5^. 62% of the data was collected using a vasopressor agent, 80% using an inotropic agent, and 11% using vasodilators. The SVRI value was lower in the male population (Men: 1826.19 ± 474.19 vs. Women: 2315.24 ± 439.85, *p* = 0.05), a difference not observed in RRI (Men: 0.92 ± 0.12 vs. Women: 0.97 ± 0.10, *p* = 0.27).

Table 2 shows the bivariate analysis in relation to the cutoff point of 1,1 RRI. The average SVRI in patients with RRI ≤ 1,1 was 1953,1$\pm$468,17 din-seg-m^2^/cm^5^ and the average SVRI in patients with RRI > 1,1 was 2120,79$\pm$673,48 din-seg-m^2^/cm^5^ (*p* = 0,62). No significant differences were found in either group for any of the hemodynamic variables, core temperature, or use de vasoactive drugs.

**Table 1:** General characteristics of the 22 patients and the 35 hemodynamic and ultrasound measurements

*Patients (n = 22)*	
Age (Mean, $\pm$ SD)	65 $\pm$9 years
Sex n (%)	Male: 13 (59)
	Female:9 (40)
BMI (Mean, $\pm$ SD)	25.7 $\pm$ 3.4
Type of surgery n (%)	Coronary artery Bypass Graft: 10 (45)
	Valvular procedure: 6 (27)
	Mixed procedure: 6 (27)
Peripheral arterial disease	5 (22)
Hemodynamics measured (*n* = 35)	
Hemodynamic variables (Mean, $\pm$ SD)	CO: 4.89 (1.22) L/min
	CI: 2.73 (0.64) L/min/m^2^
	MAP: 77.42 (10.81) mmHg
	CVP: 12.94 (3.2) mmHg
	sPAP: 37.02 (8.99) mmHg
	dPAP: 20.37 (6.03) mmHg
	mPAP: 27.4 (7.2) mmHg
	PAOP: 17 (4.82) mmHg
	SVRI: 1967.47 (478.33) din-seg-m^2^/cm^5^
RRI (Mean, $\pm$ SD)	0.94 (0.11)
Temperature (Median, IQR)	36.4 (36.2-35.5) º C
Vasopressor n (%)	22 (62)
Inotropic n (%)	28 (80)
Vasodilator n (%)	4 (11)

SD: Standard deviation, BMI: Body mass index, CO: Cardiac output, CI: Cardiac Index, MAP: Mean arterial pressure, CVP: Central venous pressure, sPAP: Systolic pulmonary artery pressure, dPAP: Diastolic pulmonary artery pressure, mPAP: Mean pulmonary artery pressure, PAOP: Pulmonary artery occlusion pressure, SVRI: Systemic vascular resistance index, RRI: radial resistance index, IQR: Interquartile range

**Table 2:** Bivariate analysis for the radial resistance index with a 1.1 cutoff point

	RRI ≤ 1.1 (*n*=32)	RRI > 1.1 (*n*=3)	*p* value
CO (Mean $\pm$ SD)	4.9 (1.26)	4.3 (0.41)	0.44
CI (Mean $\pm$ SD)	2.7 (0.66)	2.4 (0.26)	0.34
MAP (Mean $\pm$ SD)	77.6 (10.59)	75.3 (15.53)	0.73
CVP (Mean $\pm$ SD)	12.9 (3.6)	13 (3)	0.97
SVRI (Mean $\pm$ SD)	1953.1 (468.17)	2120.79 (673.48)	0.62
sPAP (Mean $\pm$ SD)	37.28 (9.13)	34.33 (6.65)	0.59
dPAP (Mean $\pm$ SD)	20.71 (6.18)	16 0.6 (2)	0.27
mPAP (Mean $\pm$ SD)	28.0 (7.2)	20 (2)	0.06
PAOP (Mean $\pm$ SD)	17.06 (5)	16.33 (2.5)	0.8
Temperature (Mean $\pm$ SD)	36.5 (0.5)	36.4 (0.3)	0.75
Norepinephrine *n*=22 (Mean ± SD)	0.17 (0.10) *n*=20	0.05 (0.02) *n*=2	0.11
Milrinone *n*=21 (Mean ± SD)	0.357 (0.12) *n*=19	0.562 (0.26) *n*=2	0.056
Vasopressin *n*=8 (Mean ± SD)	2.7 (0.7) *n*=7	2.0 (0) *n*=1	0.41
Dobutamine *n*=4 (Mean ± SD)	2.5 (0) *n*=4	NA *n*=0	NA
Nitroglycerin *n*=4 (Mean ± SD)	0.20 (0.05) *n*=4	NA *n*=0	NA
Levosimendan *n*=3 (Mean ± SD)	0.1 (0) *n*=3	NA *n*=0	NA

RRI = radial resistance index, CO: Cardiac output, CI: Cardiac Index, MAP: Mean arterial pressure, CVP: Central venous pressure, SVRI: Systemic vascular resistance index, sPAP: Systolic pulmonary artery pressure, dPAP: Diastolic pulmonary artery pressure, mPAP: Mean pulmonary artery pressure, PAOP: Pulmonary artery occlusion pressure

**Fig. 3:**
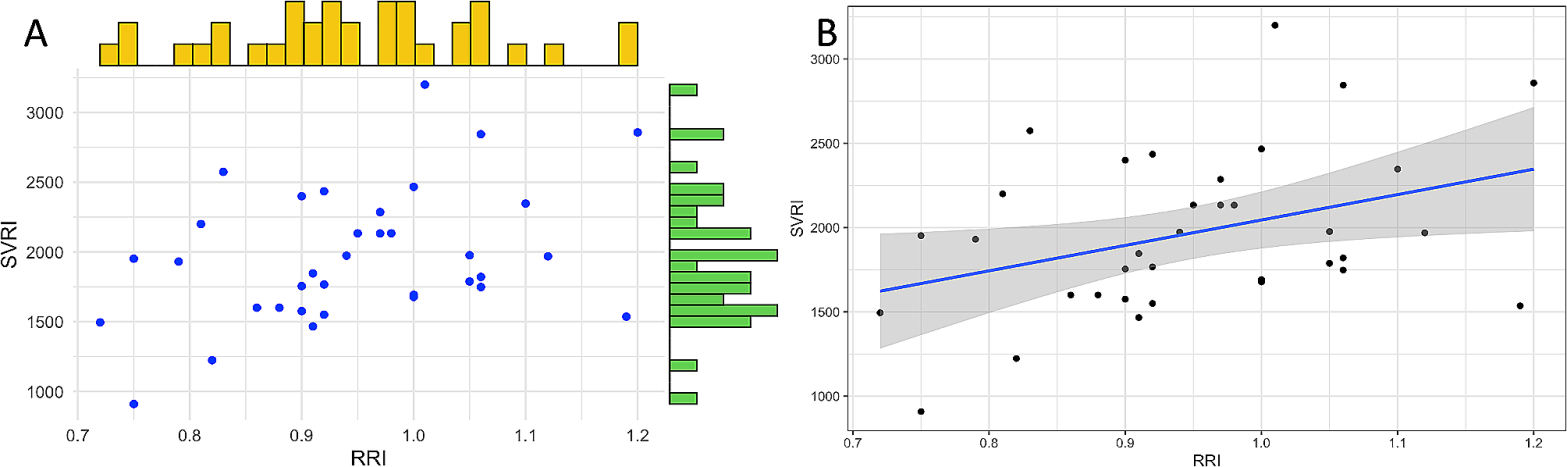
**A**. Scatter plot of the radial resistance index (RRI) in the X axis and the systemic vascular resistance index (SVRI) in the Y axis. **B**. Regression line

In a linear regression model with SVRI as the dependent variable and RRI as the independent variable, the intercept is 538.0, and the slope is 1507.0 (*p* = 0.027). Figure 3A illustrates this model; in this scatter plot, the relationship between SVRI and RRI is shown, indicating wide dispersion. Figure 3B displays the regression line of the model, depicting a slightly positive relationship between these variables. However, the variation in RRI only explains 13% of the variation in SVRI. The correlation between SVRI and RRI using the Pearson method was low (r² 0.37; *p* = 0.04; CI 95% 0.045–0.62).

**Fig. 4:**
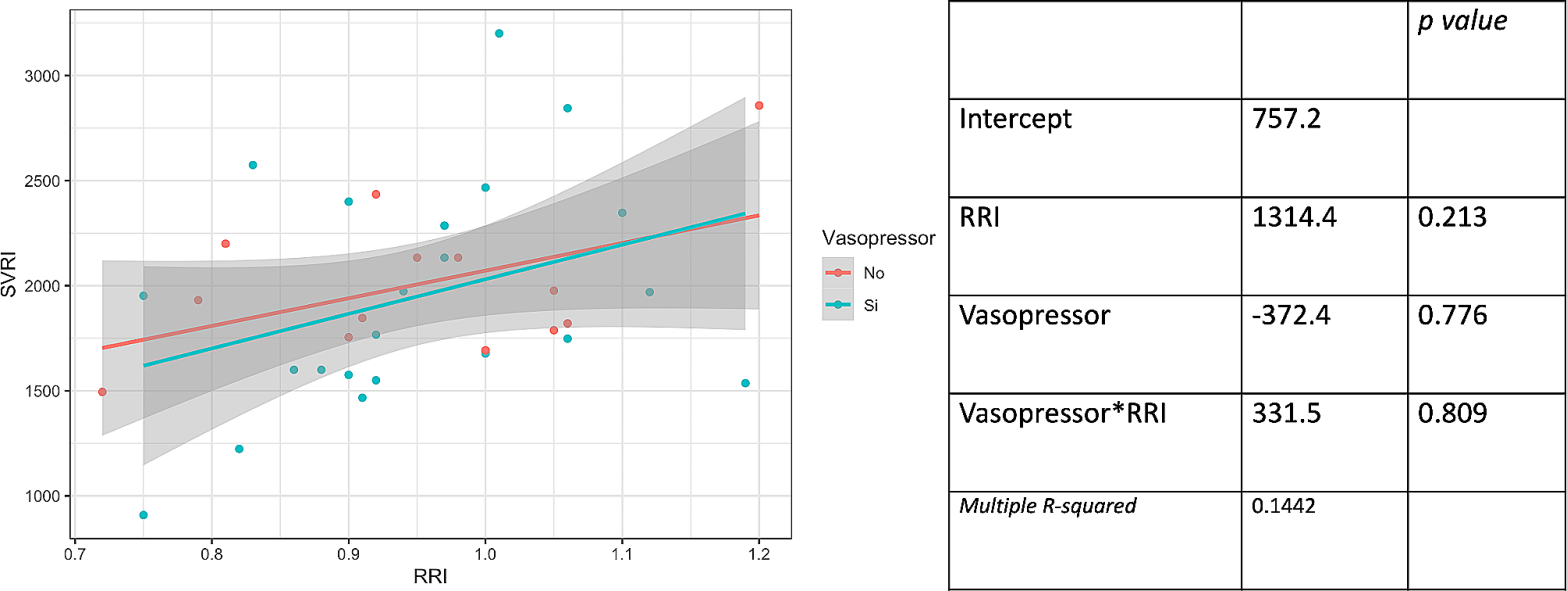
Multiple linear regression model with systemic vascular resistance index (SVRI) as the dependent variable, examining the interaction between radial resistance index (RRI) and the use of vasopressors as independent variables. Scatter plot (left) and coefficients (right) of the model

**Fig. 5:**
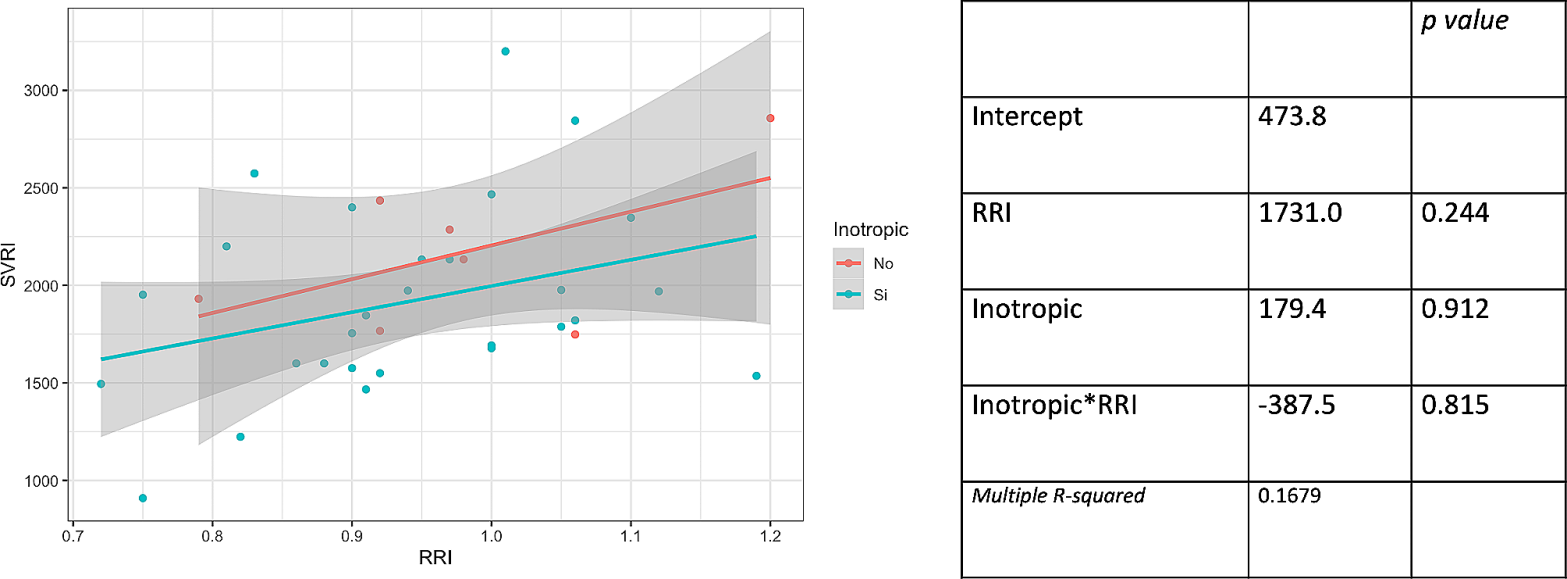
Multiple linear regression model with systemic vascular resistance index (SVRI) as the dependent variable, examining the interaction between radial resistance index (RRI) and the use of inotropes as independent variables. Scatter plot (left) and coefficients (right) of the model

Figure 4 presents the multiple linear regression model with SVRI as the dependent variable, examining the interaction between RRI and the use of vasopressors as independent variables. No interaction is observed between the use of vasopressors and RRI. Figure 5 illustrates the multiple linear regression model with SVRI as the dependent variable, exploring the interaction between RRI and the use of inotropes as independent variables. No interaction is observed between the use of inotropes and RRI.

**Fig. 6:**
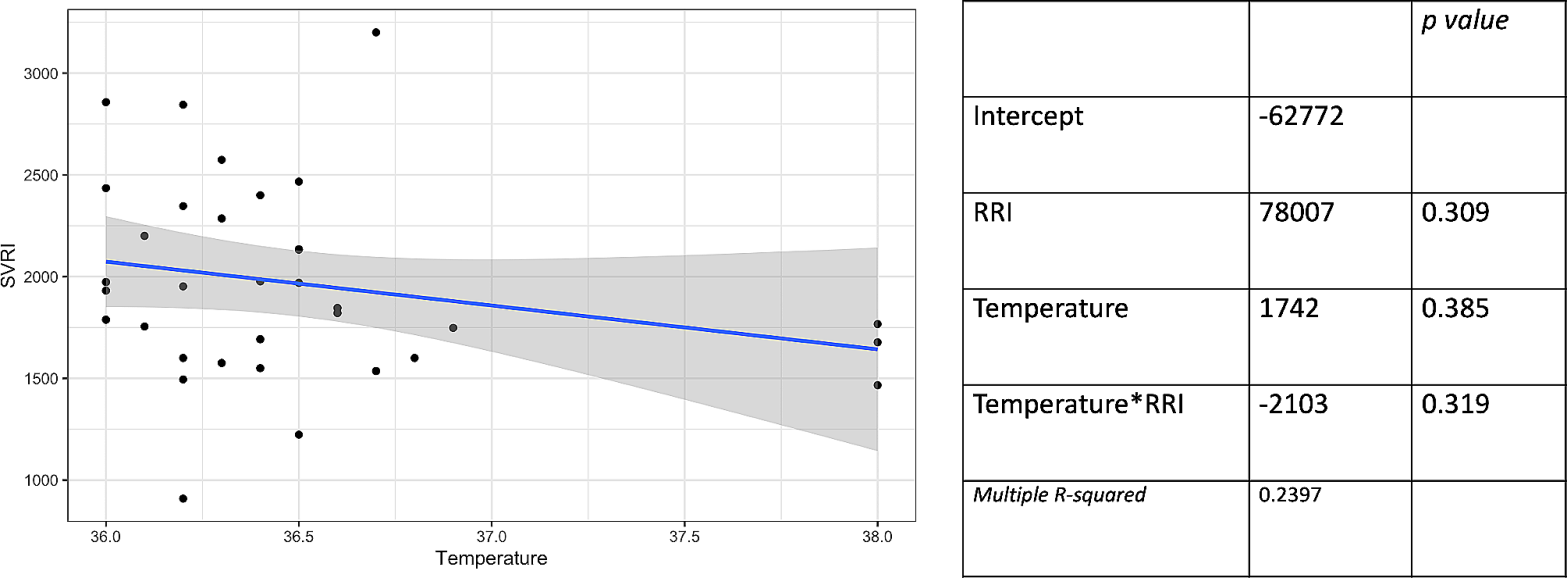
Scatter plot (left) of simple linear regression model with systemic vascular resistance index (SVRI) as the dependent variable and core temperature as independent variable. Coefficients of the multiple linear model (right) with SVRI as dependent variables and radial resistance index (RRI) and core temperature as independent variable

Figure 6 displays the simple linear regression model with SVRI as the dependent variable and core temperature as the independent variable, without an association between these variables. Additionally, the coefficients of a multiple regression model are presented, demonstrating no interaction between temperature and RRI.

**Fig. 7:**
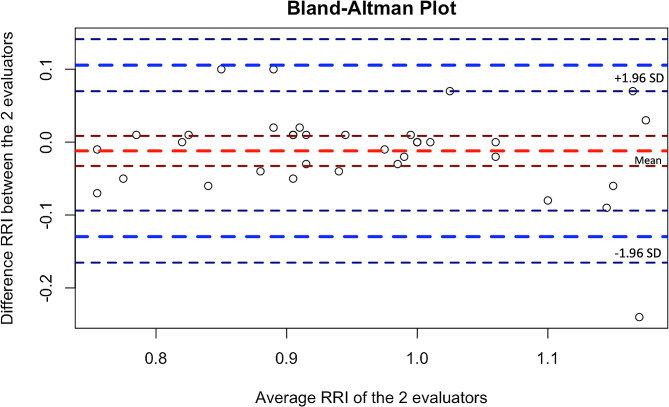
Bland-Altman plot. Dashed red line shows the mean difference. Dashed blue lines show the limits of agreement. RRI = radial resistance index

Consistency between evaluators showed an intraclass correlation coefficient of 0.88 IC (0.78–0.93). In Fig. 7, the Bland-Altman plot is shown. In this plot, we observe that the mean difference (dashed red line) is -0.012, indicating that the difference between the measurements of the RRI by the two evaluators is small. We also see the limits of agreement (dashed blue lines), the lower limit is -0.129 and the upper limit is 0.105, these limits establish the range in which approximately 95% of the differences between the data from one evaluator and the other will fall. In this graph, we can visually assess that there is good agreement between both evaluators.

## Discussion

The ultrasound assessment of SVR is not validated. However, based on physiological models proposing arterial compliance and elastance theory as major SVR determinants, different approaches based on the RI have been proposed. The RI can be obtained using pulsed Doppler examination on various arterial structures based on the application of the Pourcelot index [14].

The RI is an objective parameter indicating the amount and velocity of flow towards a tissue. Using pulsed Doppler technique on vascular structures, the maximum systolic and diastolic velocities can be determined (Vmax - Vmin), and the RI formula can be solved as (Vmax - Vmin) / Vmax [15]. This examination can be performed on any arterial vascular bed with an acceptable correlation with SVR measurement [13, 16]. Early experimental models concluded that the RI could be associated with SVR [14, 17]. In 2005, Ban et al. compared the behavior of the RI and SVRI correlation in experimental models and in patients in cardiac surgery POP, showing that the correlation varies in in vivo models (*r* = 0.98 vs. *r* = 0.58). Additionally, they showed how the insonation angle strongly interferes with the strength of correlation [13].

In 2019, Lee et al. compared the SVRI measured using PAC with the RRI measured in the anatomical snuffbox in septic patients, and establishing a strong correlation between an RRI ≤ 0,97 with an SVR < 1700 din-seg-m^2^/cm^5^, as well as a RRI < 1,1 with a SVR < 2400 din-seg-m^2^/cm^5^ [12]. In 2021, the RRI was used in a sepsis resuscitation protocol, where patients with an RRI measurement < 0,9 were assumed as patients with a low SVR. Vasopressor agents were administered early, achieving a lower volume of crystalloids and a shorter hospital stay [18].

Unlike what has been reported in the literature [12, 13, 17, 18], in this study the correlation between the SVRI and the RRI is low, with wide data scattering, limiting its use in the clinical practice. However, SVRIs in this study were higher than those previously presented [12, 13]. Ban et al. reported SRVIs no higher than 1528 din-seg-m^2^/cm^5^ [13], while the studies by Lee et al. and Devia-Menendez were conducted on patients with distributive shock for whom a low SVRI is assumed [12, 18].

In patients with vasoplegia due to septic shock, distal arteries have been described to exhibit altered elastic properties associated with changes in pulsatility and resistance indexes [19]. Likewise, early SVR and RRI correlation studies using experimental models are consistent with this premise, since it has been shown that the more compliant vessels are, the higher the correlation between the SVRI and the RRI is [17]. This suggests that patients with marked vasoplegia may experience greater alterations in peripheral RIs (including RRI), accounting for the difference in this study compared to previous research.

In this study, the use of vasoactive agents and temperature did not alter the association between SVRI and RRI. Based on these findings and previous evidence [12, 13, 17, 18], it appears that a low RRI (≤ 1.1) is a good predictor of vasoplegia in the clinical context of septic shock. However, if the predominant shock mechanism is not vasoplegia but involves multiple shock mechanisms or if there is no shock, the behavior of the elastic properties of distal vessels is unpredictable, and the RRI does not show a strong correlation with SVRI.

Ultrasound measurement is known to be dependent on the skill of the observer and their ability to reproduce the measurement method with the lowest number of variations; this becomes even more important when the change in estimation magnitude varies by a few decimal places. Flow estimation using pulsed Doppler requires the flow direction to be parallel to the insonation angle. Any insonation angle in ultrasound assessments must be under 30° to obtain a reliable measurement [20]. In this study, all the measurements were made with no need to correct the insonation angle and at the same depth, which is why it is considered a standardized measure. The intraclass coherence coefficient and the Bland-Altman analysis among different evaluators showed a good aggrement for the application of this measurement.

The study being conducted at a single site in patients whose hemodynamic profiles had SVRIs close to normal is considered a study limitation. Nonetheless, the sample size is appropriate to determine the correlation magnitude and to conclude that the RRI is not a useful measure to identify SVR changes. A selection bias occurred, so its use is not recommended to approach shock patients.

## Conclusions

In postoperative cardiac surgery patients, the correlation between SVRI measured by PAC and RRI in the anatomical snuffbox is low. Therefore, using RRI as an SVRI estimator is not recommended in this context. However, RRI measurements in the anatomical snuffbox are reproducible when conducted by trained personnel.

## Data Availability

The data used in the present study are available upon request to the corresponding author.

## Abbreviations

RRI: Radial resistance index
SVRI: Systemic vascular resistance index
PAC: Pulmonary artery catheter
POP: Postoperative
SVR: Systemic vascular resistance
CI: Cardiac Index
RI: Resistance index
SD: Standard deviation
IQR: Interquartile range
CO: Cardiac output
MAP: Mean arterial pressure
CVP: Central venous pressure
sPAP: Systolic pulmonary artery pressure
dPAP: Diastolic pulmonary artery pressure
mPAP: Mean pulmonary artery pressure
PAOP: Pulmonary artery occlusion pressure
TBSA: Total body surface area
mmHg: Millimeters of mercury
